# A generative two-stage semantic intermediary framework for explainable mental health early warning in higher education

**DOI:** 10.3389/fpsyt.2026.1866163

**Published:** 2026-07-03

**Authors:** Jianmeng Ye, Zhou-Jie Shen, Baozhen Li, Wen-Jing Yan

**Affiliations:** 1 Hangzhou Normal University, Hangzhou, Zhejiang, China; 2 Zhejiang College of Security Technology, Wenzhou, Zhejiang, China; 3 School of Mental Health, Wenzhou Medical University, Wenzhou, China; 4 Wenzhou University of Technology, Wenzhou, Zhejiang, China; 5 Zhejiang Provincial Clinical Research Centre for Mental Health, Affiliated Kangning Hospital, Wenzhou Medical University, Wenzhou, China

**Keywords:** digital health implementation, digital phenotyping, ecological validity, explainable AI, large language models, premature medicalization, university mental health

## Abstract

The psychological well-being of university students is an important public health concern and a growing implementation challenge for digital health systems. Cross-sectional psychometric screening is limited by temporal lag, selective self-disclosure, and the difficulty of distinguishing transient contextual disruption from clinically meaningful deterioration. Although digital phenotyping and predictive artificial intelligence (AI) have advanced mental health monitoring, real-world deployment in universities remains constrained by intrusive data collection, limited auditability, automation bias, and the risk that routine behavioral variation will be prematurely medicalized. In response to these implementation and governance challenges, this article proposes the Generative Semantic Intermediary Framework (GSIF), a behavior-first framework for explainable mental health early warning in higher education. GSIF is organized around three layers: ecologically feasible multimodal observation, two-stage generative semantic translation, and constrained review prioritization. Large language models (LLMs) are used not as autonomous diagnostic agents but as bounded semantic intermediaries: first translating heterogeneous institutional signals into plain-language descriptions of observable behavioral change, and then mapping these descriptions to cautious, reviewable symptom-related descriptors within established psychopathological frameworks. The framework emphasizes data minimization, role-bounded access, human-in-the-loop (HITL) verification, and explicit escalation thresholds. By making the pathway from routine data to review recommendations more transparent, GSIF offers a testable digital health architecture for earlier, more proportionate, and more governable student support workflows. Future work should evaluate its feasibility, acceptability, reviewer calibration, false-positive burden, and incremental value over existing screening and monitoring approaches.

## 1 Introduction

The psychological well-being of university students is a public health concern and an increasingly important problem for digital health implementation. As students transition into higher education, they may encounter interacting academic, social, and environmental stressors that increase vulnerability to psychological distress. Despite growing institutional investment in counseling services, many identification workflows still rely primarily on periodic screening and self-report psychometric instruments (1). Empirical work indicates that mental health-related behaviors can fluctuate over time in student populations (2) and that cutoff-based instruments may misestimate prevalence in nonclinical populations (3, 4). In many institutions, scales are administered intermittently, sometimes only once at academic entry. This cross-sectional snapshot can reduce sensitivity to short-term behavioral changes associated with elevated risk and may be poorly aligned with the dynamic nature of student life. Machine learning approaches have recently been applied to improve the efficiency and stratification of standard screening instruments, though they remain constrained by the same fundamental dependence on self-report input (5).

To complement static scales and reduce temporal lag, researchers and campus administrators have increasingly explored digital phenotyping and predictive artificial intelligence (AI). Smartphone and behavioral-signal studies suggest these approaches can help characterize changing risk-related trajectories and, in some settings, support earlier review than periodic screening alone (2, 6–8). At the same time, real-world deployment raises practical, epistemological, and ethical challenges that remain only partially resolved.

First, some digital phenotyping paradigms rely on data sources that are clinically informative but operationally difficult to sustain at campus scale. Architectures requiring continuous physiological monitoring (e.g., wearable heart-rate variability) or pervasive affect sensing (e.g., continuous facial analysis) may face substantial implementation barriers. In decentralized university ecosystems, these high-intrusion modalities can increase privacy concerns, complicate compliance with data minimization and legal-governance requirements, raise infrastructure costs, and reduce long-term participation (9, 10).

Second, many AI architectures for psychological early warning remain predominantly data-driven and can be difficult to interpret at the level required for clinical decision support. Systematic reviews of passive monitoring approaches note substantial methodological heterogeneity and limited consensus on how low-level behavioral features should be clinically interpreted (11), while broader medical AI literature continues to highlight challenges in transparency and justification of model outputs (12). Several analyses have specifically examined the challenges facing AI-based mental disorder recognition, noting that current systems face fundamental limitations related to symptom subjectivity, ecological validity of training data, and the gap between statistical performance and clinical utility (13). In campus environments, behavior is often context-sensitive. Similar patterns may also occur during non-pathological periods, such as examination weeks, highlighting the need for stronger contextual mediation before symptom-level inference.

This article argues that effective and ethically robust early warning systems should not rely exclusively on correlation-based prediction and may benefit from digital health architectures that make the behavior-to-symptom pathway more explicit. Rather than functioning as isolated score generators, predictive systems may be more actionable when designed to approximate hierarchical, evidence-based clinical reasoning while remaining bounded by implementation safeguards. The central aim is therefore not to propose an autonomous diagnostic system, but to describe a transparent, behavior-first framework that can be empirically evaluated in future campus digital health workflows.

This conceptual article is organized around three guiding questions. First, how can routine, low-intrusion campus data be used without treating passive institutional traces as direct evidence of psychopathology? Second, how can a digital early-warning workflow make the transition from observable routine change to review recommendation more transparent and auditable? Third, what governance and human-review safeguards are needed to reduce premature medicalization and automation bias in university mental health support? The central argument is that a useful campus early-warning architecture should be behavior-first, semantically staged, and review-oriented. The remainder of the article first reviews the empirical and conceptual basis for this design, then presents the three-layer GSIF architecture, discusses its clinical and governance implications, and finally outlines a staged evaluation agenda for future empirical work.

### 1.1 Related work and empirical basis for the design rationale

Prior work relevant to GSIF can be grouped into three areas. The first is digital phenotyping and passive monitoring, where smartphone, wearable, and institutional signals have been used to characterize longitudinal variation in mental-health-related functioning. This literature supports the value of temporal and ecological signals, but it also shows substantial heterogeneity in data sources, missingness, engagement, and clinical interpretation. The second area is explainable and human-in-the-loop (HITL) AI. Studies of automation bias and clinical decision support indicate that interpretable outputs alone are insufficient unless they are embedded in workflows that preserve human judgment and make escalation criteria visible. The third area is governance-oriented digital health design, including data minimization, role-bounded access, audit logging, and proportionality. GSIF integrates these strands by treating explainability as a property of the whole workflow: what data are collected, how they are semantically translated, who can view which outputs, and how review decisions are constrained.

The design rationale of GSIF is motivated by convergent empirical signals in the current literature. First, screening workflows in university and other nonclinical populations remain vulnerable to underreporting and calibration problems, including concealment bias and prevalence misestimation when cutoff-based instruments are used without contextual interpretation (2–4). These findings support longitudinal, context-enriched evidence streams alongside one-time psychometric assessments.

Second, campus-scale deployment constraints are empirically visible in studies on digital phenotyping and data governance: higher-intrusion sensing and broad data collection increase implementation burden, privacy risk, and compliance complexity, especially under modern legal regimes such as PIPL (9, 10). Prospective college-focused digital phenotyping studies also report data-quality constraints (e.g., missingness and variable engagement), reinforcing feasibility-first system design (7).

Third, the interpretability literature indicates that performance metrics alone are insufficient for clinical adoption. Evidence from automation bias and clinician-AI workflow studies suggests that staged presentation, oversight, and explicit bias-mitigation strategies are important for the safe use of model outputs (14–17). Mental-health-specific XAI frameworks similarly emphasize that deployment-facing understandability requires both process transparency and interpretable output presentation (18). GSIF operationalizes this evidence via a behavior-first translation stage before symptom-level risk recommendation and frames explainability as a workflow property rather than a *post hoc* technical add-on.

Psychiatric assessment can often be conceptualized as a hierarchical, graded inferential process. In routine practice, clinicians typically infer depression from multiple observations rather than directly observing a single unitary construct. They collect observations of daily functioning, contextualize these facts within the student’s lived reality, aggregate them into symptom-level descriptors (e.g., chronic insomnia, psychomotor slowing, social withdrawal), and then estimate syndromal risk (19). This graded inference view also aligns with modern nosological paradigms such as the Hierarchical Taxonomy of Psychopathology (HiTOP), which conceptualizes mental health as a dimensional, hierarchical spectrum rather than rigid binary categories (20).

**Figure 1:**
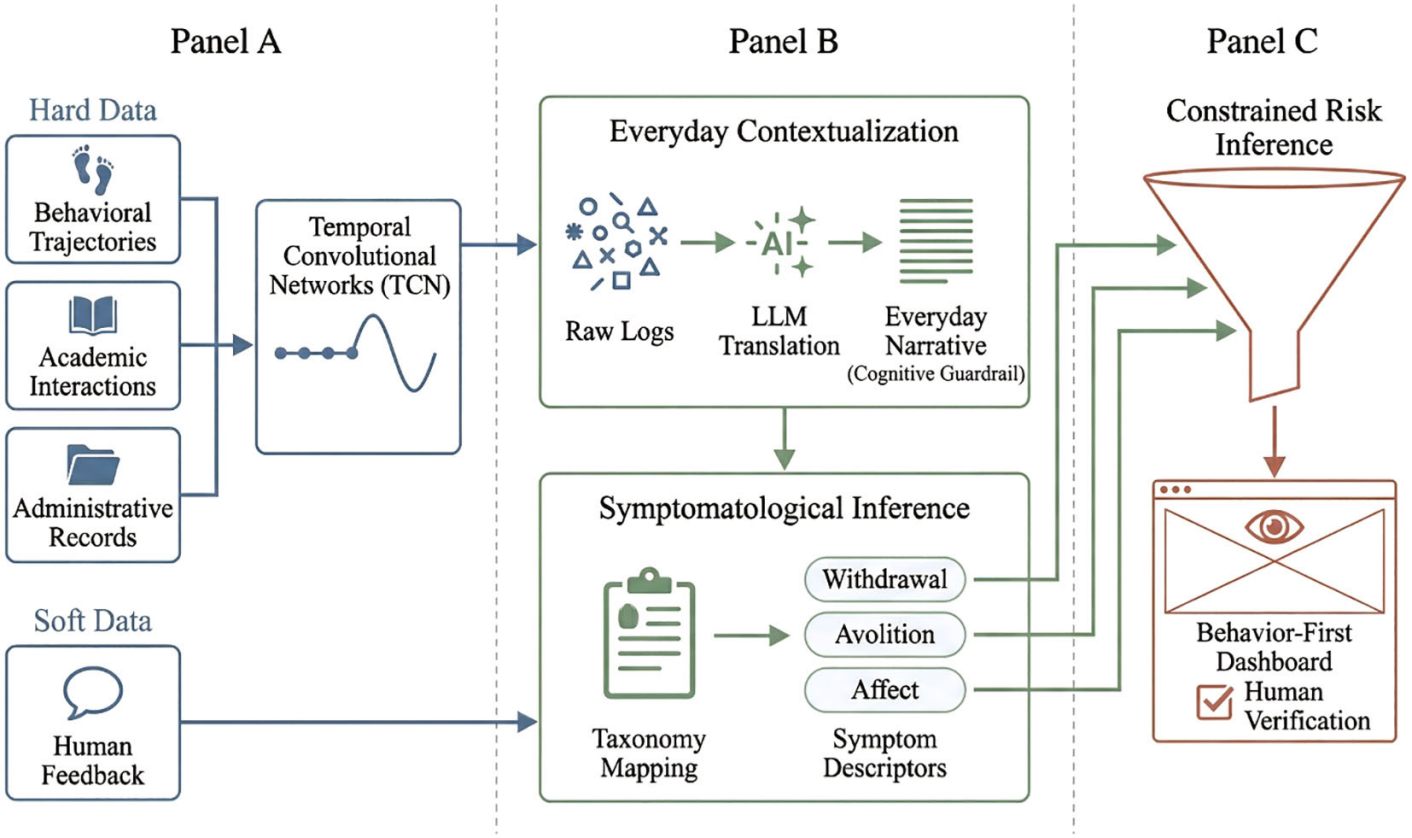
Overview of the generative semantic intermediary framework (GSIF). **(A)** Evidence layer: integration of institutional hard data and human feedback through temporal encoding; **(B)** Intermediary layer: two-stage LLM translation from raw logs to everyday narratives and subsequent symptomatological inference; **(C)** Decision layer: constrained risk stratification and behavior-first dashboard for HITL verification.

To computationalize this clinical workflow and address translational bottlenecks, we propose the GSIF. Building on prior interpretable and hybrid approaches, GSIF decomposes prediction into a tripartite design (1): Ecologically Valid Multimodal Observation, (2) Two-Stage Generative Semantic Translation, and (3) Constrained Risk Inference (see **Figure 1**). By prioritizing feasible campus data and requiring explicit behavioral contextualization before symptom-level mapping, GSIF aims to improve interpretability and reduce unjustified risk escalation.

The framework can be expressed as a staged transformation rather than a single predictive function. Let X denote low-level ecological traces and structured observations, B denote a behavior-first narrative, S denote bounded symptom-related descriptors, and R denote a review-prioritization output. GSIF constrains the workflow as X -> B -> S -> R. The first transition summarizes observable routine change without psychiatric labeling. The second transition maps behavior-first descriptions to cautious, candidate descriptors only when convergence, persistence, and contextual plausibility are present. The final transition produces a review priority rather than a diagnostic classification. This notation is intended to clarify the logic of the framework, not to specify a validated mathematical model.

## 2 The generative semantic intermediary framework

### 2.1 Layer 1: ecologically valid multimodal observation (the evidence layer)

Rather than depending primarily on highly intrusive sensing modalities, this layer harmonizes data across four institutionally feasible pillars, integrating both objective digital traces (hard data) and structured human observations (soft data).

#### 2.1.1 Behavioral trajectories (hard data)

This category encompasses passive mobility and routine metadata extracted from ubiquitous campus infrastructure, rather than behavioral observations in the psychological sense. Key fields include access control frequencies, smart card dining transactions, and campus Wi-Fi connectivity logs. These records indicate spatial-temporal participation patterns, such as presence in particular campus zones or the timing of routine transactions, but they do not directly observe motivation, affect, intention, or clinically meaningful conduct. Within GSIF, these fields are therefore treated as low-level ecological traces that may help characterize deviations from an individual routine baseline. Any psychological interpretation requires contextualization through subsequent semantic and human-review layers.

#### 2.1.2 Academic interactions (hard data)

This data stream captures observable platform interaction metadata within educational management systems, including login frequency, dwell time, video completion, and quiz response behavior. These fields should not be interpreted as direct measures of attention, motivation, or cognitive engagement. A student may leave a video early because of disengagement, prior mastery, technical problems, competing obligations, or external stressors. The construct-validity problem is therefore central: academic platform traces can support descriptions of participation patterns, but they cannot establish internal cognitive states. GSIF uses these signals only as behavior-adjacent indicators requiring semantic contextualization and human verification.

#### 2.1.3 Administrative records (hard data)

Administrative records are semantically underdetermined unless interpreted within a theory-guided and context-sensitive framework. A leave request, absence from a check-in, or interruption in an enrollment-related process does not indicate psychological distress by itself. Such records may reflect illness, family obligations, academic scheduling, financial constraints, or many other nonclinical circumstances. In GSIF, administrative records are therefore used only as limited contextual indicators of possible disruption in routine institutional functioning. Their interpretation is constrained by a graded inference view of assessment: low-level institutional facts may contribute to a broader review hypothesis only when they converge with other observable changes, persist over time, and remain plausible after benign explanations are considered.

#### 2.1.4 Human feedback (soft data)

The Evidence Layer may integrate human feedback in two separable forms. The first is structured observational coding, such as predefined categories for markedly reduced verbal responsiveness, substantially slowed speech pace, repeated withdrawal from group activities, or unusually limited eye contact during routine interactions. These codes should be role-appropriate, brief, and linked to observable interactional features rather than diagnostic judgments. The second is short contextual notation by trained personnel, used only to record relevant benign explanations or immediate support concerns. For replicability, GSIF should prioritize structured codes as machine-readable inputs and treat free-text notes as restricted contextual material subject to stricter access control and human review.

The heterogeneous fusion of ecologically valid streams is intended to establish a multi-dimensional baseline that can complement self-report limitations, including potential concealment. This evidence matrix provides the empirical foundation for the generative semantic translation in the subsequent layer.

### 2.2 Layer 2: the two-stage semantic intermediary layer (generative clinical translation)

The core methodological innovation of the GSIF resides in the Semantic Intermediary Layer. Recent end-to-end deep learning systems have produced meaningful gains in risk prediction performance; however, systematic reviews and conceptual analyses indicate that translational challenges remain when low-level behavioral signals are mapped directly to high-level psychiatric labels without an explicit intermediate interpretive step (11–13). Under these conditions, models may over-attribute context-dependent behaviors (e.g., exam-period routine disruption) to severe psychopathology, which can increase false positives and contribute to premature medicalization (3).

To address these limitations while building on prior progress, the GSIF introduces a structured semantic bridge rather than a purely single-step mapping pipeline. Specifically, it draws on the natural-language reasoning capacity of large language models (LLMs) to support a constrained two-stage translation under explicit cognitive guardrails, consistent with the emerging literature on bounded human-LLM collaboration and mental-health-related text structuring (21, 22).

#### 2.2.1 Sub-layer 2a: everyday behavioral contextualization

Raw digital logs and fragmented administrative records, such as spatial movement frequencies, meal transaction timestamps, and academic leave requests, do not by themselves carry clinical meaning. A sensor indicating that a smart card was not used at the dining hall records only an absence of transaction at that location and time. It does not, on its own, distinguish among benign schedule variation, religious practice, temporary physical illness, financial constraint, or a possible change in social or motivational routine.

In this initial translational stage, the generative LLM functions as a structured interpreter of heterogeneous evidence rather than a diagnostic engine. Its purpose is to synthesize Layer 1 inputs into cohesive, plain-language accounts of observable changes in daily routine, participation, or engagement. At this stage, the model should be explicitly restricted from assigning psychiatric labels, estimating disorder likelihood, or implying that any single behavioral deviation has clinical significance.

For example, the co-occurrence of “zero dormitory gate exits over 72 hours,” “two recent sick leave submissions,” and “zero dining hall transactions” may be rendered as: “The student’s recent records suggest reduced out-of-room activity, disruption of usual meal-related routines, and temporary interruption of academic participation.” This form of everyday contextualization is intended to operate as a cognitive guardrail, translating high-dimensional institutional traces into behaviorally interpretable descriptions that remain open to multiple explanations before any symptom-related mapping is considered, consistent with broader calls to keep LLM-supported clinical workflows collaborative and bounded (23).

#### 2.2.2 Sub-layer 2b: bounded symptom-related mapping

Once behavior-first summaries have been generated, this sub-layer supports a more bounded form of symptom-related interpretation. Operating as a clinical decision-support component, the LLM considers the Sub-Layer 2a narratives together with relevant human observations from Layer 1, such as an advisor noting reduced verbal engagement or a peer reporting withdrawal from routine group activities. The aim is not to infer diagnosis from routine campus data, but to organize whether a pattern may warrant closer human review using familiar descriptive frameworks.

At this stage, the model may map integrated behavioral profiles to cautious, candidate descriptors drawn from established frameworks such as DSM-5-TR or the dimensional spectra of HiTOP (20). These outputs should be treated as provisional, low-confidence semantic aids for review, not as determinations that a student is experiencing a defined symptom or disorder. In practice, the model should privilege broad, behavior-linked descriptors, such as reduced social participation, possible disruption of daily rhythm, or decreased academic engagement, and should avoid pathologizing normative stress responses or context-specific routine changes. Any escalation beyond this interpretive layer should depend on trained human assessment and institutionally defined governance criteria.

By separating everyday contextualization from bounded symptom-related mapping, GSIF aims to keep the intermediate semantic space interpretable, reviewable, and less prone to premature medicalization. .

### 2.3 Layer 3: the decision layer (constrained review prioritization)

The final tier of the architecture is the Decision Layer, which is designed to support review prioritization rather than autonomous risk determination. Because Sub-Layer 2b has already converted heterogeneous observations into structured, bounded, and reviewable descriptors, the final step is not diagnosis but a constrained triage judgment about whether additional human attention may be warranted. In this framing, outputs are better understood as review flags or support priorities, such as “routine monitoring,” “follow-up suggested,” or “clinical review may be warranted,” rather than definitive classifications of mental state.

Rather than relying exclusively on unconstrained pattern recognition from noisy raw data, the Decision Layer evaluates combinations of behavior-linked descriptors under explicit institutional and clinical guardrails. This design may improve auditability by making clear which observed patterns contributed to a recommendation and which escalation thresholds were applied. In university settings, where severe adverse outcomes are low-base-rate events and behavioral features are often high-dimensional and context-sensitive (7, 11), such constraints may help limit overfitting and reduce unwarranted alert escalation (14, 15). Even so, any output from this layer should remain non-diagnostic, contestable, and subject to trained human review before any contact, referral, or intervention decision is made.

HITL verification should be triggered by explicit criteria, including persistence of the pattern beyond a short transient window, convergence across more than one data stream, absence of an obvious benign explanation, data completeness sufficient for review, and proportionality between the concern and the proposed response. The Decision Layer should therefore support staged escalation: routine monitoring when evidence is weak or transient, contextual follow-up when observable disruption persists, and qualified clinical review only when convergent patterns and human contextual assessment indicate that further evaluation may be warranted. These criteria are intended to make escalation auditable and contestable rather than automatic.

## 3 Framework advantages and clinical applicability

### 3.1 Logical reasoning and auditable two-stage evidence chains

One recurring limitation of conventional deep learning models in psychiatric applications is that their probabilistic outputs (e.g., an 85% risk score) can be difficult to translate into actionable reasoning. In the ethically sensitive context of university mental health, probability estimates are often more useful when paired with interpretable evidence chains. GSIF therefore employs LLM-based reasoning modules to produce structured intermediate explanations before final review recommendations.

Crucially, the LLM is computationally constrained to execute the semantic translation sequentially. Rather than outputting a solitary risk metric, the system generates a transparent, top-down evidence chain. For instance, a review-facing dashboard could present the following structured rationale to a qualified reviewer (**Box 1**).

Box 1 **Step-by-step generative reasoning chain for behavior-first review support.**

Step 1: Ecological Evidence (Layer 1). The system identifies a recent pattern that may merit review, such as no dormitory gate exits over 72 hours, no dining hall transactions during the same interval, a recent sick leave submission, and an advisor note documenting reduced eye contact during a routine interaction.

Step 2: Everyday Contextualization (Sub-layer 2a). The LLM translates these fragmented records into a plain-language summary: *"Recent records suggest reduced out-of-room activity, disruption of usual meal-related routines, temporary interruption of academic participation, and lower-than-usual interpersonal engagement."*

Step 3: Bounded Symptom-Related Mapping (Sub-layer 2b). If permitted within the review workflow, the system may organize this pattern using cautious, candidate descriptors drawn from established frameworks, such as *reduced social participation*, *possible disruption in daily routine*, and *reduced interpersonal engagement*. These descriptors are provisional and intended only to support human interpretation.

Step 4: Review-Oriented Decision Support (Layer 3). The Decision Layer may then recommend an action level such as continued monitoring, contextual follow-up, or qualified clinical review, depending on the persistence, convergence, and contextual plausibility of the observed pattern. No autonomous diagnosis or intervention is made, and all outputs remain subject to human verification.

By requiring articulation of everyday behavioral context before symptom labeling, the framework is intended to support more auditable hypotheses prior to intervention and to make subsequent human review more transparent (16).

From an evidence standpoint, this sequencing should be treated as a testable implementation hypothesis: compared with direct score-only dashboards, staged explanations may improve human review quality and reduce automation-biased acceptance of unsupported alerts. Existing work on explainability, automation bias, and clinician-AI workflow design provides partial empirical support for this expectation (14–17).

### 3.2 Proactive early warning: fusing temporal dynamics with generative translation

In some students, periods of heightened distress may be preceded by longitudinal behavioral change rather than a single abrupt event. Accordingly, a campus support system can benefit from modeling when concerning trajectories are emerging. To capture temporal structure in behavioral metadata, GSIF integrates temporal convolutional networks (TCNs) at the Evidence Layer before generative semantic translation.

TCNs use dilated causal convolutions to capture long-range temporal dependencies in numerical behavioral metadata. Rather than treating a single missed meal as decisive evidence, the model captures progressive multi-week shifts in dining regularity and mobility relative to each student’s historical baseline. When temporal anomalies are detected (the “when” and “how much”), the representation is passed to the two-stage LLM semantic layer for contextual interpretation (the “what” and “why”). The generative translation from ecological campus data to review-relevant interpretive descriptors is summarized in **Table 1**.

**Table 1:** The evidence matrix: generative translation from ecological campus data to review-relevant interpretive descriptors.

Data category	Raw institutional fields (layer 1)	Sub-layer 2a: everyday behavioral contextualization	Sub-layer 2b: bounded symptom-related mapping
Behavioral trajectories	Access control frequencies (dormitory/library); dining payment times and frequency; Wi-Fi connection durations by zone	“He recently stays in his dorm constantly without leaving.”/”She has exclusively eaten at 2:00 a.m. for five consecutive days.”	Possible reduction in social participation; possible disruption in daily routine or sleep-wake regularity; nonspecific slowing or disengagement requiring contextual review
Academic interactions	Academic portal login frequency and dwell time; online learning platform video completion rates and quiz responsiveness	“He opens his class schedule but closes it immediately.”/”She exits video lectures after 10 minutes and leaves all questions blank.”	Possible reduced academic engagement; possible difficulty sustaining attention or task persistence; behavior pattern requiring human contextual assessment
Administrative records	Formal leave request timing and frequency; recorded absences from required administrative check-ins; repeated short-term interruptions in routine enrollment-related processes	“The student has recently submitted multiple formal leave requests and has had repeated interruptions in routine administrative participation.”	Possible stress-related burden or temporary functional disruption; contextual factor for review rather than symptom evidence on its own
Human feedback	Advisor interview codes (e.g., eye contact < 3s); peer observer reports (e.g., refuses group work)	“He doesn’t look up or make eye contact when speaking.”/”She actively refuses all collective activities and group invitations.”	Possible reduced interpersonal engagement; possible blunted or constrained observable affect; behavior pattern warranting review, not stand-alone clinical inference

The GSIF structures evidence hierarchically. Raw institutional records are first translated into plain-language descriptions of observable behavioral change that serve as cognitive guardrails, and only then into cautious, candidate symptom-related descriptors. These descriptors are intended for decision support and human review only. They are not diagnoses, should not be treated as proof of psychopathology, and must be interpreted in light of local context, alternative explanations, data limitations, and institutional governance boundaries.

This neuro-symbolic fusion, combining temporal encoding with generative reasoning, may expand the practical window for supportive review and follow-up. Empirical studies using multisource and longitudinal modeling in student populations suggest that temporally sensitive behavioral analysis can help characterize concerning change trajectories and support earlier, more proportionate review in some settings (24, 25).

### 3.3 Incremental validity and the mitigation of premature medicalization

To justify the additional computational and infrastructural costs of a generative two-stage framework, studies should test whether it adds incremental predictive or operational value relative to simpler screening and monitoring baselines (26). A key concern in mental health AI is premature medicalization: models over-labeling developmentally normative distress (e.g., exam-period stress and temporary social withdrawal) as severe psychopathology. Such misclassification can increase false positives, contribute to alert fatigue, and weaken practitioner trust (3). Evidence from university stigma-intervention trials indicates that help-seeking behavior is context-dependent, underscoring the need to interpret algorithmic risk signals within local psychosocial context rather than as stand-alone clinical truth (27).

Accordingly, future evaluations should report endpoint-specific gains rather than aggregate discrimination alone, including false-positive burden, alert precision under low-base-rate conditions, time-to-actionable-review, and inter-rater agreement in human verification. This evaluation strategy is consistent with the broader view that implementation utility depends on both incremental performance over existing workflows and the reliability of the surrounding decision process (3, 17, 26).

### 3.4 Testable propositions for future evaluation

Because GSIF is proposed as a digital health framework rather than a validated clinical instrument, its value should be evaluated through explicit, falsifiable propositions. **Table 2** summarizes key testable propositions, comparators, expected advantages, and potential endpoints that can guide future feasibility studies, simulation experiments, expert review panels, and prospective implementation research in university mental health systems.

**Table 2:** Testable propositions and evaluation endpoints for the GSIF.

Proposition	Comparator	Expected advantage	Possible evaluation endpoint
Two-stage semantic translation will reduce premature symptom labeling.	Direct mapping from raw behavioral data to risk scores.	More cautious interpretation and fewer unjustified escalations.	False-positive burden; proportion of alerts judged clinically or supportively appropriate.
Behavior-first narratives will improve human reviewer calibration.	Risk dashboards that present symptom labels or probability scores first.	Better contextual verification and less automation-biased acceptance.	Inter-rater agreement; reviewer confidence calibration; override rate.
Role-bounded output will improve governance compatibility.	Unified dashboards exposing all users to the same data and interpretations.	Reduced inappropriate access to sensitive symptom-related information.	Access-log audit findings; privacy acceptability; governance compliance review.
Ecologically feasible campus data will improve implementation sustainability.	High-intrusion sensing such as continuous physiological or facial monitoring.	Lower participation burden and better alignment with data minimization.	Student acceptability; data missingness; retention; implementation cost.

## 4 Ethics, privacy, and human-AI collaboration

The transition toward more continuous, low-burden campus support infrastructures raises substantial ethical questions. Although GSIF is proposed as a more transparent and auditable alternative to opaque end-to-end prediction, the use of routine behavioral data in university settings still requires strict limits on scope, access, interpretation, and downstream action. For that reason, GSIF should be understood not as a surveillance model, but as a governance-dependent decision-support framework whose acceptability depends on data minimization, role-bounded access, and meaningful human accountability.

### 4.1 Privacy and data governance

Operationalizing any form of continuous behavioral monitoring in higher education creates a persistent tension between earlier support identification and students’ rights to privacy, autonomy, and informational self-determination. Under regulations such as China’s Personal Information Protection Law (PIPL), processing behavioral and health-adjacent data requires careful attention to lawful basis, necessity, proportionality, and transparency (10). These requirements are especially important in nonclinical educational environments, where the existence of administrative data does not by itself justify expansive secondary use for mental health-related inference. Broader structural challenges in open mental health data governance, including tensions between declared openness and practical access barriers, further underscore the importance of designing transparent and genuinely accountable data-sharing architectures from the outset (28).

To improve legal and ethical alignment, institutions considering GSIF should adopt strict, multilayer governance boundaries. First, data minimization should limit inputs to routine, low-intrusion metadata that are demonstrably necessary for narrowly defined support triage purposes, while excluding content surveillance, off-campus personal data, and any collection of private communications. Monitoring the semantic content of emails, messages, calls, coursework, or social media should remain outside system scope (9). Second, access should be role-based and purpose-limited, with clear separation between nonclinical staff, clinical personnel, and technical administrators. Nonclinical staff should not receive symptom-level outputs, inferred disorder labels, or unrestricted access to raw longitudinal records. Third, deployment should prioritize secure local control, audit logging, retention limits, and documented review pathways so that students’ data are not exposed to unnecessary third-party processing or function creep.

### 4.2 “Behavior-First” HITL integration

A central concern in AI-supported mental health workflows is over-automation of interpretive and intervention decisions. GSIF therefore adopts a HITL model in which generative components serve only as bounded decision-support tools (23). The system does not diagnose, does not determine intervention on its own, and does not displace professional judgment. Responsibility for interpretation, contextual assessment, and any subsequent action remains with appropriately trained human personnel, with the highest-stakes decisions reserved for appropriately trained clinical professionals where applicable (29).

To operationalize this principle, GSIF uses a behavior-first HITL interface with explicit governance boundaries for different user roles. One practical advantage of the two-stage semantic structure is that it allows frontline university personnel to review observable behavioral summaries without being asked to interpret psychiatric labels. In this model, nonclinical staff, such as academic advisors or student affairs personnel, may be permitted to view only Sub-Layer 2a style descriptions of routine disruption or reduced engagement, and only for the limited purpose of checking context, documenting benign explanations, or routing concerns through established support channels. Symptom-related mappings, triage recommendations, and any escalation toward mental health services should be restricted to qualified reviewers under institutional protocol.

Upon an initial alert, the interface should therefore present everyday behavioral context first, for example: “Recent records suggest reduced class attendance, fewer canteen visits, and lower participation in routine interactions.” This presentation keeps the first review focused on observable change rather than implied pathology. Reviewers should be expected to consider alternative explanations, data completeness, timing, and known situational stressors before any higher-level interpretation is shown. Access to Sub-Layer 2b outputs and Decision Layer recommendations should occur only within a staged review workflow designed to reduce automation bias, preserve human discretion, and ensure that any support response is proportionate, documented, and contestable (17).

## 5 Implementation and evaluation agenda

A key next step is to move GSIF from a conceptual framework to an empirically assessed digital health workflow. Initial work should not begin with autonomous risk deployment. Instead, evaluation should proceed through staged, low-risk studies that test whether the framework improves interpretability, proportionality, and governance compared with simpler screening or score-based monitoring approaches.

First, feasibility studies should examine whether institutionally available data can be transformed into reliable behavior-first summaries without expanding surveillance beyond a narrowly defined support purpose. Relevant endpoints include data completeness, missingness, cost of integration, reviewer workload, and the frequency with which alternative benign explanations are identified during human review. Second, acceptability studies with students, counselors, academic advisors, and data-governance personnel should assess whether the framework is perceived as transparent, proportionate, and contestable. These studies should pay particular attention to whether students understand the difference between behavioral support flags and diagnostic inference.

Third, simulation and vignette-based experiments can test whether staged behavior-first explanations reduce automation bias. Reviewers could be randomized to receive either direct risk scores, symptom-label-first outputs, or GSIF-style sequential narratives, with outcomes including inter-rater agreement, escalation appropriateness, confidence calibration, and false-positive escalation. Fourth, prospective pilot studies should assess incremental value over existing periodic screening workflows by measuring alert precision, time-to-actionable-review, support-routing accuracy, and potential unintended harms such as alert fatigue or perceived surveillance. Across all stages, evaluation should include auditability, fairness, and role-based access outcomes, because implementation success in this context depends as much on governance and human workflow as on predictive performance.

Future empirical evaluation should proceed in stages. A feasibility study should first document the available data fields, missingness, temporal coverage, and governance constraints in participating institutions. A simulation or vignette study should then compare GSIF-style staged narratives with direct risk scores and symptom-label-first dashboards. Relevant outcomes include reviewer calibration, inter-rater agreement, escalation appropriateness, false-positive burden, and time-to-actionable-review. A prospective pilot should compare GSIF against existing periodic screening and routine monitoring workflows, with predefined baselines and endpoint-specific metrics. Ablation analyses should examine the contribution of each design component, including removal of the behavior-first narrative stage, removal of structured human feedback, and replacement of staged review with direct risk-score presentation. These studies are necessary before GSIF can be treated as a validated clinical or operational instrument.

## 6 Discussion

The GSIF proposes an explainable, behavior-first digital health architecture for mental health-related decision support in higher education. By introducing an intermediate semantic layer between routine institutional data and review recommendations, the framework aims to make the pathway from observed behavioral change to human assessment more transparent, auditable, and governable. Its two-stage translation is intended to keep interpretation grounded in everyday behavioral context before any bounded symptom-related mapping is considered.

GSIF is therefore best understood as a non-diagnostic support framework that may complement, but not replace, periodic screening, clinical evaluation, and established campus support processes. Its value will depend on empirical validation, strict governance, limited data scope, and meaningful human oversight. Under those conditions, GSIF offers a testable pathway for digital health systems to explore earlier and more proportionate student support without treating routine campus data as stand-alone evidence of psychopathology.

The main implication of GSIF is not that routine campus data can diagnose mental disorder, but that digital early-warning systems require an auditable interpretive space between low-level institutional traces and human action. This distinction is especially important in higher education, where routine variation is common and the cost of false escalation may include stigma, mistrust, and perceived surveillance. The framework also has practical limitations. It requires local governance capacity, role-bounded access control, reviewer training, careful calibration of escalation criteria, and empirical testing across diverse campus contexts. Its temporal modeling components may add complexity and should be justified only if they improve review quality or support timing over simpler baselines. GSIF should therefore be understood as a testable conceptual architecture whose value depends on future evidence, not as a ready-to-deploy clinical system.

Several limitations should be acknowledged. The framework is proposed at the conceptual level and has not yet been empirically tested. The feasibility of real-time deployment in diverse campus environments, the acceptability to students with varying privacy expectations, and the calibration of LLM-generated behavioral narratives against human clinical judgment all remain open empirical questions. The neuro-symbolic integration of TCNs and LLMs, while theoretically coherent, introduces implementation complexity that must be weighed against simpler alternatives. Furthermore, the generalizability of the framework beyond Chinese university contexts requires cross-cultural validation given the specific regulatory environment (PIPL) and campus infrastructure assumptions embedded in the design.

Despite these limitations, GSIF contributes to the growing literature on responsible AI in mental health by shifting emphasis from predictive performance metrics to the transparency and auditability of the behavior-to-symptom mapping pathway. As digital health systems increasingly operate in ethically sensitive educational environments, frameworks that make their reasoning explicit, their scope bounded, and their escalation thresholds observable may offer a more governable alternative to black-box prediction. The testable propositions outlined in **Table 2** provide a structured agenda for moving from conceptual design to empirical evaluation.
